# The Welsh School of Social Service

**Published:** 1920-08-28

**Authors:** 


### The Welsh School of Social Service.

Medical officers had a good innings at the Welsh School of Social Service, which has concluded a successful week's session at Llandrindod Wells. The problems surrounding the treatment of venereal disease were discussed at length, and Dr. Colston Williams, the Glamorgan Medical Officer of Health, urged women to make the removal of this evil a main plank in their platform. Dr. Pole, Radnorshire's medical officer, rather startled the gathering by announcing that there was no venereal disease in his county!

Dr. Llewellyn Williams, M.C., F.R.C.S., senior medical officer of the Ministry of Health, having emphasised the need for an extension in Wales of maternity and child welfare work, and especially the provision of more maternity hospitals, pointed out that national health depends to a great extent on national character, and that venereal disease, intemperance and night clubs are moral questions. Hygiene, he said, must be taught and applied in schools, colleges, cinemas, and churches. People should have less faith in drugs, Dr. Williams declared, and more in nature.

